# The revival of telemedicine in the age of COVID-19: Benefits and impediments for Pakistan

**DOI:** 10.1016/j.amsu.2021.102740

**Published:** 2021-08-21

**Authors:** Maheera Farooqi, Irfan Ullah, Muhammad Irfan, Anab Rehan Taseer, Talal Almas, Mohammad Mehedi Hasan, Fatima Muhammad Asad Khan, Abdulaziz Alshamlan, Abdulaziz Abdulhadi, Vikneswaran Raj Nagarajan

**Affiliations:** a Department of Internal Medicine, Dow University of Health Science, Karachi, Pakistan; b Kabir Medical College, Gandhara University, Peshawar, Pakistan; c Hayatabad Medical Complex, Peshawar, Pakistan; d Royal College of Surgeons in Ireland, Dublin, Ireland; e Department of Biochemistry and Molecular Biology, Faculty of Life Science, Mawlana Bhashani Science and Technology University, Tangail, 1902, Bangladesh; f Dow University of Health Sciences, Karachi, Pakistan

Dear Editor

Defined as “the use of information and telecommunication technologies (ICT) in medicine, telemedicine intends to provide appropriate healthcare at a distance, hence eliminating the need for direct contact between a patient and physician [1]. It can be classified according to the type of interaction (pre-recorded or real-time) and type of format in which information is conveyed (videos, pictures, audio, etc.) [2]. Particularly in the setting of a natural or man-made disaster, telemedicine is known to function as a key component in the emergency response, enabling people to access routine care and health support despite widespread disruptions in health services [3].

The relevance of telemedicine to our health systems is more evident than ever today as we continue to battle the COVID- 19 pandemic that has modified our lifestyle and approach to medical care. In the face of lockdowns and social distancing protocols, telemedicine technologies are being employed for online consultations, monitoring and evaluating symptoms, tracking and circumventing COVID-19 hotspots, and addressing individual concerns through chat bots [4].

Although the age of COVID-19 has significantly propelled the adoption of telemedicine services globally, its market was booming even prior to the onset of the COVID-19 pandemic, with a market size estimated around US$50 billion as of 2019, projected to increase over 9-fold in the coming decade [5]. A growing body of literature supports the role of telemedicine in providing timely, affordable, and premium quality healthcare services surpassing geographical barriers, which is especially advantageous for resource limited countries. However, while it is being integrated in the health infrastructure in USA, Europe and South East Asia with increasing momentum, its future in the developing world remains obscure [6].

Although the rate is considerably slower than developed countries, developing countries are gradually adapting to the changing times with efforts to make high-quality healthcare accessible to the masses from the comfort of their residence via digital interventions. Sub-Saharan Africa, for example, has reported a significant increase in mobile health technology [7]. The implementation of telemedicine amid a concomitant burden of communicable and non-communicable diseases in low and middle income countries (LMICs) can have consequential impacts in addressing the basic health needs of the population. By reducing travel costs and time, telemedicine enables rural and marginalized communities to access the same quality of medical resources and care as urban dwellers, and promotes health equity [6].

With the widespread use of smartphones, SMS messaging, and automated calls, telemedicine is a vital medium to provide adequate care for chronic illnesses such as diabetes and hypertension, infectious diseases, and pediatric and reproductive health in LMICs to limit inpatient care, monitor adherence, encourage self-care and improve health outcomes [[8], [9], [10]]. Furthermore, the telemedicine revolution has been beneficial for surgical patients with timely follow-up and detection of time-sensitive surgical site infections [11], and in the treatment and prevention of mental health issues in LMICs [12].

Since COVID-19 has resulted in a subsequent spike of communicable diseases in the developing world [[13], [14], [15], [16]], telemedicine will have play a critical role in optimizing patient care while reducing hospital visits and mitigating disease transmission.

Like most LMICs, there is a lack of a well-structured healthcare system in Pakistan where only 3.2% of the current GDP is being invested in the health sector [17]. A densely populated country with an alarming physician to population ratio of 1:1000 which is suboptimal according to World Health Organization, the medical resources in the country were stretched further at the crest of the COVID-19 outbreak, which presented an ideal opportunity for the revival of telemedicine.

Telemedicine is not an alien concept for Pakistan; it has benefited the country in the past and has a promising future. It has been functioning in Pakistan for many years on a small scale, however, following the COVID-19 pandemic, there has been an appreciable shift in healthcare delivery with a focus on improving digital healthcare services as experts report a staggering growth of 900% in telemedicine companies [18]. Apart from private enterprises, multiple initiatives have also been taken by the government, with the formation of a telemedicine COVID-19 web portal and mobile applications to provide free online consultations [19].

Since its introduction in 2003, several telemedicine programs have been carrying out successful digital healthcare interventions for years [18], and have served the community relentlessly during the first and second waves of COVID-19 in Pakistan. The department of Telemedicine at King Edward Medical University provides care via mobile phones, landline telephones, and Skype. In light of the COVID-19 crisis, their Corona Help Desk and virtual OPDs have been quite successful. Sehat Kahaani, a telemedicine start-up that connects home-based female physicians to underserved communities via mobile applications and e-health clinics to carry out consultations and follow-ups has proved to be another popular venture. In the past, telepsychiatry has been used to serve communities impacted by natural calamities [20]. More recently, Agha Khan University Hospital initiated Telemental Health services, which provide services to patients via video videoconferencing to tackle the psychological impacts of the COVID-19 pandemic [21]. Additionally, a mobile application called Teeku has previously been used to catalyze routine childhood immunization rates in rural settings which kept an electronic record of the vaccination status of the child, while also sending follow-up reminders via text messages [22].

Presently, telemedicine is being extensively employed in the on-going COVID-19 mass immunization campaign in Pakistan with user-friendly websites e.g., NIMS website, and SMS services available in both English and Urdu for areas that do not have access to the internet [23]. However, other LMICs incorporating innovative strategies, such as the utilization of mobile health technology in the mass immunization campaign for oral cholera vaccine in rural Haiti, serve as a potent reminder that there is still room to improve and explore the untapped potential of the telemedicine landscape in Pakistan [24].

Although the aforementioned services have had an unquestionable impact, telemedicine in Pakistan is still in its developing stages and is not conveniently accessible to the masses. A variety of limitations have impeded its widespread use. Poverty and low literacy rate of the population are the major contributors in perpetuating mistrust in the communities [25]. A dearth of resources and experts, high infrastructure costs, geographical barriers, and the disparity between urban and rural areas based on limited internet coverage and mobile ownership are some additional barriers [26].

According to a recent survey, 98.2% of doctors working in the public health sector in the city of Karachi reported a lack of training workshops or conferences for telemedicine, which is indicative of sufficient knowledge gaps and inadequate training among healthcare workers in this field [27].

Although telemedicine is witnessing a new dawn in the post COVID-19 era, its integration in the Pakistan's health system mandates dedicated efforts from all stakeholders. Proper education and training in this field are a necessity along with patient engagement. Since telemedicine programs cannot be expanded by solely relying on individual physicians, it is imperative to devise national guidelines and policies to manage telemedicine facilities on a routine basis. More investment is critical, and funds should be allocated in developing the infrastructure for extensive delivery of high-speed internet even during adverse weather conditions. It is important to take initiatives intending to make cell services and high-speed internet available in far-flung areas of the country. There is a need for proper laws and regulations to safeguard data privacy and provide ethical delivery. To expedite immunization programs in the country, user-friendly mobile apps can be a favorable intervention for registration, locating vaccination centers, and surveilling possible adverse reactions. Vaccination centers should be equipped with telemedicine facilities to keep records of any missed vaccinations. Lastly, the government should consider the idea of integrating a block-chain on the supply of COVID-19 vaccination to ensure adequate delivery and storage and minimize the risk of the supply being stolen or smuggled.

In conclusion, telemedicine is an asset for the health system of any country and has the potential to alleviate the burden on the understaffed hospitals of Pakistan. In the past, it has immensely helped in tackling disparity in access to high-quality healthcare between different regions of the country with the ever-increasing population, fragile healthcare infrastructure, financial constraints, and deficiency of well-qualified doctors and equipment. The COVID-19 pandemic has created an unprecedented opportunity for Pakistan to address these issues by broadening the use of telemedicine indefinitely. Although the challenges hindering its widespread use are multi-fold, increased federal investment and appropriate training of medical professionals in the telemedicine sector can accelerate its integration as a mainstream constituent in the healthcare infrastructure of Pakistan.

## Ethics statement

The present study includes printed and published information; therefore, the formal ethical clearance was not applicable for this study.

## Author contribution

MF, IU, MI: conceived the idea, designed the study, and drafted the manuscript. ART, TA, MMH: conducted literature search and created the illustrations. FMAK, AA: revised the manuscript critically and refined the illustrations. AA and NRN: revised the final version of the manuscript critically and gave the final approval.

## Consent

NA.

## Registration of research studies

Name of the registry: NA.

Unique Identifying number or registration ID: NA.

Hyperlink to your specific registration (must be publicly accessible and will be checked): NA.

## Guarantor

Talal Almas, RCSI University of Medicine and Health Sciences, 123 St. Stephen’s Green, Dublin 2, Ireland, Talalalmas.almas@gmail.com, +353834212442.

## Funding

None.

## Declaration of competing interest

The authors declare that there is no conflict of interests.
